# Tungsten Based Spectrally Selective Absorbers with Anisotropic Rough Surface Texture

**DOI:** 10.3390/nano11082018

**Published:** 2021-08-07

**Authors:** Niloufar Pirouzfam, Kursat Sendur

**Affiliations:** Faculty of Engineering and Natural Sciences, Sabanci University, Istanbul 34956, Turkey; npirouzfam@sabanciuniv.edu

**Keywords:** rough surface, spectrally selective absorber, emissivity, anisotropic rough surface, optical properties, extreme environments, thermo-mechanically stable materials, refractory metals

## Abstract

Spectrally selective absorbers have received considerable interest due to their applications in thermophotovoltaic devices and as solar absorbers. Due to extreme operating conditions in these applications, such as high temperatures, thermo-mechanically stable and broadband spectrally selective absorbers are of interest. This paper demonstrates anisotropic random rough surfaces that provide broadband spectrally selective absorption for the thermo-mechanically stable Tungsten surfaces. Anisotropic random rough surface has different correlation lengths in the x- and y-directions, which means their topography parameters have directional dependence. In particular, we demonstrate that spectral absorptance of Tungsten random rough surfaces at visible (VIS) and near-infrared (NIR) spectral regions are sensitive to correlation length and RMS height variations. Our results indicate that by optimizing random rough surface parameters, absorption values exceeding 95% can be obtained. Moreover, our results indicate that anisotropic random rough surfaces broaden the bandwidth of the high absorption region. It is shown that in VIS and NIR regions, the absorption enhancements of up to 47% and 52% are achieved for the isotropic and anisotropic rough surfaces, respectively.

## 1. Introduction

Thermal solar [1,2,3], solar thermophotovoltaic [4], nuclear fusion [5,6,7], and aerospace applications [8] rely heavily on materials capable of operating at high temperatures. These materials must be able to operate at temperatures exceeding 1500 °C [9]. Tungsten (W) is increasingly being used for different applications, such as solar devices, due to its outstanding chemical and thermal stability, high melting point, wear-resistance and the ability to store energy for over long periods of time [10,11,12]. Moreover, Tungsten is one of the refractory metals with intrinsic absorption in the VIS to NIR region that operates at high temperatures for the applications in which a high absorption is needed. Tungsten has high absorption in the visible range, but its large real part of the dielectric constant leads to a high reflection in the infrared regime. This causes flat films to have 60% or less absorption [13]. Tungsten can be textured in the shape of a pyramid micro/nanostructures to boost absorption [14]. Detailed modal analysis predicted that periodic Tungsten absorbs light in a wide angular range. However, oxidation effects result in low stability of textured Tungsten at high temperatures [14].

In the literature, various methods to control the absorption and emission of light from surfaces have been demonstrated, including photonic crystals [15,16], optical metamaterials [17,18], nanoparticles [19,20], multilayer thin films [21], and micro/nanotextured structures [22]. Since the 1970s, the principle of using surface structures and surface patterns has been used as an alternative to thin-film coatings for controlling spectral reflection and absorption. Anti-reflective treatments are used extensively in the optic industry for applications ranging from lenses, lasers, cameras, solar cells, and visible and near-infrared light systems to windows, missile domes, defense, and infrared laser systems [23,24]. In military, aerospace, and some industrial applications, which primarily utilizes infrared (IR) spectrum, reflection is a major issue. A common approach to improve optical reflection is to use several thin layers of dielectric materials that are mounted on the exterior surface of the window or optical component [25]. Enhancing photon absorption by increasing the active layer thickness is a relatively simple method, but the balance between charge and absorption also limits the thickness of the layer. One of many techniques for enhancing absorption without raising the actual layer thickness is to pursue a light attenuating structure, allowing the number of internal passes inside the functioning layer to improve the total length of the optical path [26]. At higher frequencies, the surface texture has a significant impact on the material’s interaction with the electromagnetic wave [27]. Textured or porous layers can scatter light, and hence, intensify the duration of light travel through the absorber. Recently, significant progress was made in modeling and manufacturing nanostructures to handle surface optical absorption and emission properties [19,28].

In the case of high optical power, the optical properties of material are no longer linearly related to the intensity of the incident light. One effect is nonlinear absorption, which may cause heating. This nonlinearity changes the propagation of intense light through the medium [29]. Incident light energy may be efficiently concentrated in resonant nanostructures, and this causes the temperature of nanostructure increase [30]. The activation of surface plasmon resonance results in this phenomenon, which manifests a sufficient increase in optical absorption. Light is absorbed almost entirely by free electron transitions inside the conduction band in metals [31,32]. On the optical side, heating changes a nanostructure’s optical response, which is dependent on the nanostructure’s and environment’s dielectric characteristics [33]. Optical heating can be used in various applications including water heating [34], biomedical application [35], surface coloring [36], and many other fields. On the other hand, this increase in the temperature of surface could affect the act of surface as a sensor, which should be take into consideration in sensitive applications. In the literature, the temperature dependency of the thermal expansion coefficient of water, as well as thermal boundary conductance at the interface, are taken into account [37]. Moreover, it is shown that by changing the temperature of the nano resonator, the emitted signal can be modulated [38].

Selective absorbers are commonly used in various fields due to their high absorptivity at specific wavelengths [39]. Spectrally selective filters can be used in various applications involving solar absorbers [40], sensors [41], passive cooling [21,42], and thermophotovoltaic devices [43]. Optimization of energy use could be achieved using the control of solar spectra. In addition to the consideration of cost, having the right transition wavelength can increase the efficiency of a solar absorber for specific applications significantly. Solar selective coatings are more common spectral selective absorbers studied in the literature [44]. Nanostructures can act as spectrally selective absorbers on the substrates [45]. While several studies exist in the literature regarding nanostructures’ effect on the absorption spectra, the ability of random textures to use as spectrally selective absorbers has not been studied yet. The non-periodic structures can achieve high absorption rates, with structural sizes of one micron [46].

In this manuscript, a random distribution of surface features with varying feature profiles and depth is used to obtain high absorption in Tungsten. Scattering at rough interfaces between the absorber layers causes a change in the electromagnetic field angle of incidence within the absorber layers. This leads to a phenomenon known as internal coupling, which allows light to couple inside the absorber [47]. Using this concept, randomly textured surface substrates have been successfully used for the integration into solar cells of rough interfaces [47]. Here, we use this concept for Tungsten surfaces, a stable thermomechanical material, to increase the length of the optical path in a thin absorber layer significantly. Our approach goes one more step to demonstrate broadband spectrally selective absorber using Tungsten anisotropic rough surfaces. In this case, the formation of anisotropic surface can be achieved by stretching an isotropic surface in certain directions, resulting in different lateral correlation lengths. It represents the random rough surface interface with varying correlation lengths in the x- and y-directions. We propose a technique to engineer the surface morphology based on anisotropic surface roughness for improving the surface optical characteristics. Isotropic and anisotropic random rough surfaces of Tungsten with various RMS heights and correlation lengths are investigated. The random rough surface in this study is characterized by Gaussian power spectral density (PSD) with RMS heights and correlation lengths. Surface properties of textured Tungsten surface are modeled using the finite-difference time-domain (FDTD) technique, which enables analysis of textured Tungsten surface absorbance. Using this method, a high absorption over a broad spectral band is obtained without adding any extra layers or coatings.

## 2. Materials and Methods

In this section, first, we describe the methodology to form a randomly rough surface made of Tungsten. After that, a numerical approach is applied to obtain the optical properties and spectral absorption of random rough surfaces in the wavelength range of 300–3000 nm. The procedure for obtaining optical properties from random rough surfaces is summarized. In the next step, the anisotropic random rough surface of Tungsten is examined, and the effect of this random texture on the optical properties of surface and absorbance bandwidth is reported. The data in this manuscript are obtained theoretically using rough surface interfaces described with Gaussian power spectral densities and by using a Gaussian random number generator [48]. The size of 3D grids is 10 × 10 × 10 nm^3^ in our models and the uncertainty was set to 1 × 10^−5^. Rough surfaces with Gaussian power spectral densities are common when representing a variety of interfaces in natural systems including rough metal surfaces, rough graphene surfaces, sea surface temperatures, and 2D turbulence [49].

**Figure 1:**
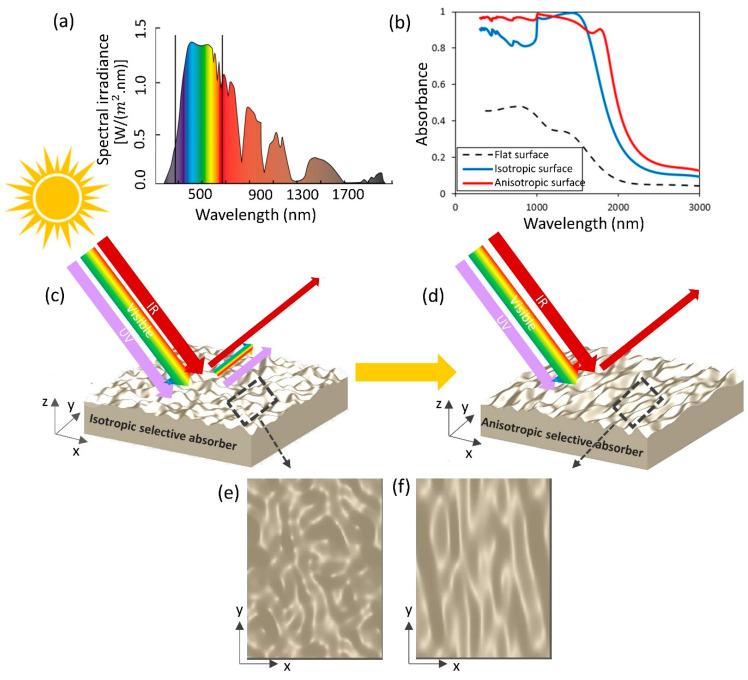
Schematic of proposed selective absorber; (**a**) solar radiation spectrum; (**b**) comparison of absorption spectrum for flat surface and rough surface; (**c**) scattering from isotropic random rough surface; (**d**) scattering from anisotropic random rough surfaces; (**e**) top view of isotropic rough surface; (**f**) top view of anisotropic rough surface.

The schematic of the proposed system is shown in Figure 1. Here, the isotropic and anisotropic random rough surface could increase the absorption in the VIS and NIR regions. Solar irradiance spectrum covers a wide range of wavelengths as shown in Figure 1a. The curve (that starts at about 300 nm) shows the incident solar power per square meter at each wavelength just above the Earth’s atmosphere. The solar spectrum is separated into three different parts including UV, visible, and infrared which is shown by a solid line in Figure 1a. Moreover, in panel (b) of Figure 1 the effect of roughness over a flat surface and the comparison of isotropic and anisotropic random rough surfaces is shown. As we will discuss later in the manuscript, the absorption spectrum increases when the surface is rough. In addition, modifying the surface anisotropy impacts the absorption especially in the visible and near-infrared regions. A schematic of isotropic and anisotropic random rough surfaces and their working principle in the scattering of light is displayed in Figure 1c,d. It is observed that all the UV and VIS light and some parts of infrared light are absorbed using anisotropic rough surfaces. Moreover, the top view of both isotropic and anisotropic random rough textures can be seen in Figure 1e,f.

Rough surfaces exhibit less reflectance and better absorption than flat surfaces [50,51]. In reality, different types of surface defects and roughness can manifest while using mechanical machining or material synthesizing processes, which result in different PSD spectra. Surface roughness is one of the important factors affecting the reflectivity/emissivity due to the incident angle change on different facets [52]. In the current study, random texture geometries are characterized by their statistical properties, height distribution, and correlation length. The height distribution specified as sigma RMS, determines the variation of heights from a smooth planar and represents the standard deviation of the distribution of surface heights. Correlation length (L_c_) defines a short distance between two points for which the heights of a rough surface are correlated with each other.

Surface roughness is quantified using PSD functions as the spread of height deviations from a mean plane and the lateral distribution/distance over which the height variation occurs. The Gaussian surface described using the Gaussian PSD function is one of the simple approximations of random surface roughness. The three-dimensional Gaussian randomness being achieved from specifying a given spectral density referring to [53]:

(1) $$W=\left(\frac{l_{x}l_{y}h^{2}}{4\pi }\right)\exp\left[-{\left(\frac{K_{x}l_{x}}{2}\right)}^{2}-{\left(\frac{K_{y}l_{y}}{2}\right)}^{2}\right],$$

where h is the root mean square (RMS), $l_{x}$ and $l_{y}$ are the correlation length along the x- and y-directions, respectively, and $K=\sqrt{{\left(k_{x}\right)}^{2}+{\left(k_{y}\right)}^{2}}$ is the wavevector in the radial direction. Moreover, $K_{x}=2\pi x/l_{x}$ and $K_{y}=2\pi y/l_{y}$. The autocorrelation function corresponding to (1) is

(2) $$\rho \left(x,y\right)=h^{2}\exp\left[-{\left(\frac{x}{l_{x}}\right)}^{2}-{\left(\frac{y}{l_{y}}\right)}^{2}\right],$$

**Figure 2:**
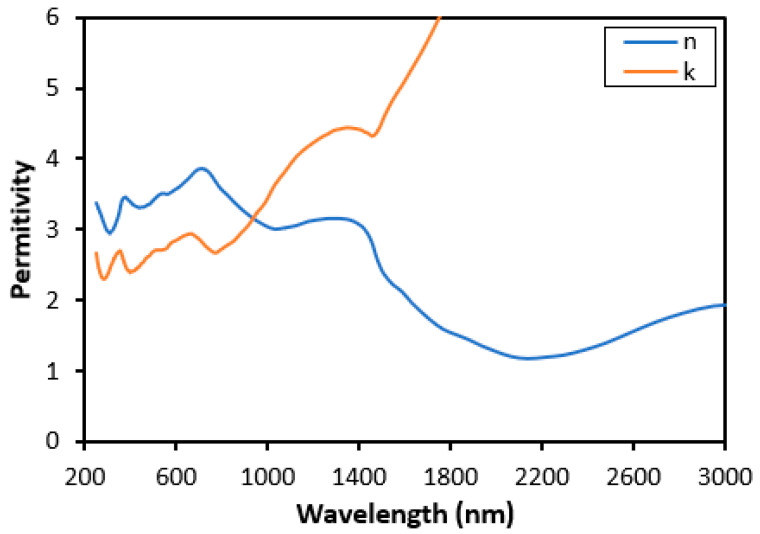
The real and imaginary part of the permittivity of the Tungsten.

To get a better understanding of the light absorption characteristics of W refractory metal, the finite difference time domain (FDTD) simulations were carried out to obtain the full-wave solution of Maxwell’s equations. The FDTD [54] approach is one of the most appealing techniques for studying light absorption from randomly formed small particles in computational electromagnetics. It produces a frequency-domain electric-field and magnetic-field distributions for all frequencies of interest. The geometry of random rough surface is inserted in the commercial simulation software (Lumerical) as a developed script based on Gaussian distribution, which is mentioned above. Our simulation is set in 3D, and the upper and bottom surfaces in the z-direction are subjected to the perfectly matched layer (PML) boundary condition, and the periodic boundary condition is applied on the side surfaces in the FDTD simulation region. A broad frequency plane wave is incident from the top of the surface with the linear polarization along the x-axis. The transmittance and reflectance monitor are set on the bottom and top sides of the surface, respectively, to collect the propagated light. The light absorption spectrum is then visualized at different absorption resonance wavelengths. Moreover, the magnetic (H) and electric (E) components of the incident electromagnetic radiation are parallel to the surface, while the wavevector k (direction of oscillation) is perpendicular to the structure. The permittivity of Tungsten is taken from Palik’s book [55] and the real and imaginary part of permittivity is illustrated in Figure 2. Under these conditions, the light reflection, transmission, and absorption properties of random rough surfaces are evaluated. The thickness of the samples is 1000 nm. For this thickness, Tungsten’s transmission in the entire spectral range is nearly zero, indicating an almost total absence of transmission. The power flow across a surface on an averaged time basis is defined by [56]:

(3) P=∫Sds=∫12Re[E×H*]ds,

in the above formula, S is the Poynting vector which is the cross product of E and H vector. E and H are the electric and magnetic field intensities, respectively. Here, $H^{*}$ denotes the magnetic field vector’s complex conjugate, and s denotes the surface area. The reflectivity is given as:

(4) $$R=\frac{P_{r}}{P_{i}},$$

where $P_{r}$ represents the reflected light’s power and $P_{i}$ represents the incident light’s power. Then, to calculate the absorption coefficient of a solar absorber, the formula below is used [57]:

(5) A(w)=1−T(w)−R(w)

where T(w) and R(w) are the frequency-dependent transmission and reflection parameters, respectively. The maximum absorption is obtained when the reflection and transmission coefficients are minimized. Since the optical properties of the material can be affected by both the intrinsic properties and the surface morphology, the study of surface engineering could be an excellent approach to change the optical properties.

Numerous studies have been conducted on the control of absorption using surface microstructures such as shallow grating [58,59,60]. One advantage of optical control via surface gratings is the high thermal stability of optical devices, which are typically fabricated on bulk materials and thus do not contain thermal discontinuities, in contrast to multiple antireflection or filtering coatings. This indicates that spectral control via surface gratings is an appealing prospect for high-temperature applications.

Inspired by the grating coupler, anisotropic random rough surfaces are taken into consideration in the next part. Anisotropic surfaces increase the absorption by acting as a more effective light trapping structure and they can act as effective passive spectrally selective absorbers. The anisotropic rough surfaces can be generated when a randomly rough surface has two different correlation lengths along x, y, marked as $l_{x}$, $l_{y}$, respectively and the reflection and absorption of the surface is affected. Tungsten is chosen as the refractory metal which can be used in high temperature and compared to noble metals such as Ag and Au, has high thermal stability.

**Figure 3:**
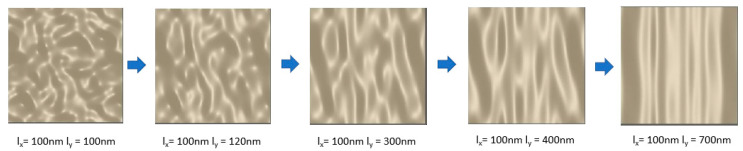
Anisotropic random roughness texture formed by random Gaussians.

The proposed technique has advantages over additional top layers. Using the proposed method as a spectrally selective device reduces the cost as no extra material are needed over the rough tungsten surface. Spectral filters with additional top layers also involve additional steps for patterning. In the proposed technique, there is no need to use any special mask for producing patterns in the lithography step to act as spectral selective filters. Additional manufacturing steps can be eliminated, which involve patterns on top of a surface as these steps need a clean room environment and different stages including deposition, lithography, and etching. On the other hand, manufacturing a random rough surface can be obtained using different methods such as powder metallurgy, direct laser deposition [61], chemical methods [62], and mechanical machining, most of which are direct methods without the need for a cleanroom environment. In addition, there are some new techniques to produce a nano-size surface roughness to be used in optical devices such as single-point diamond turning technology [63]. The top view of various correlation lengths for an anisotropic random rough surface can be seen in Figure 3. The dark grey color in the figure shows the higher heights (peaks) and the light grey shows the lower heights.

## 3. Results and Discussion

**Figure 4:**
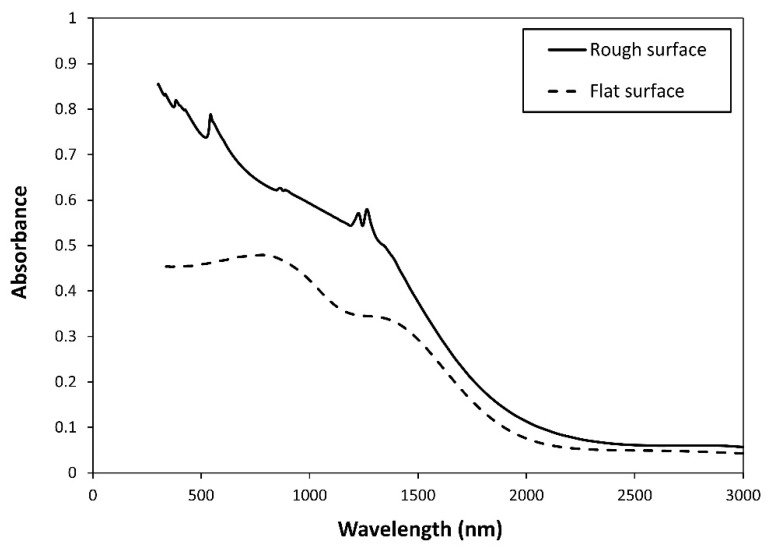
Comparing absorbance of flat and rough (l_x_ = 100 nm, l_y_ = 100 nm, and h = 50 nm) Tungsten finite thickness film by the FDTD solution.

We first studied the effect of surface roughness on Tungsten surfaces. To analyze the influence of surface morphology on spectral absorption of Tungsten surfaces in the VIS and NIR spectral regions, the absorption spectra of the flat and roughened Tungsten surface are compared in Figure 4. In this case, the rough surface is isotropic with correlation lengths $l_{x}$ = 100 nm, $l_{y}$ = 100 nm, and RMS surface height h = 50 nm. As shown in Figure 4, the spectral absorption of Tungsten increases significantly for a rough surface compared to a flat Tungsten surface, especially for wavelength below 1500 nm. This phenomenon comes from the wave interactions and the corresponding effects on absorption. It is worth noting that in the spectral region of interest, 0.4–1.5 µm, Tungsten does not support surface plasmons. Tungsten has shown a nonzero value of 2.5–4 for the real and imaginary parts of its permittivity. Within this spectral range, n decreased gradually while k increased monotonically, as previously shown in Figure 2. Please note that, the absorption reduces in the longer wavelengths, and there is not a notable difference between flat and rough surfaces. Our results indicate a strong relationship between the absorption spectra of rough Tungsten surfaces and the correlation length and RMS height of the rough surface.

**Figure 5:**
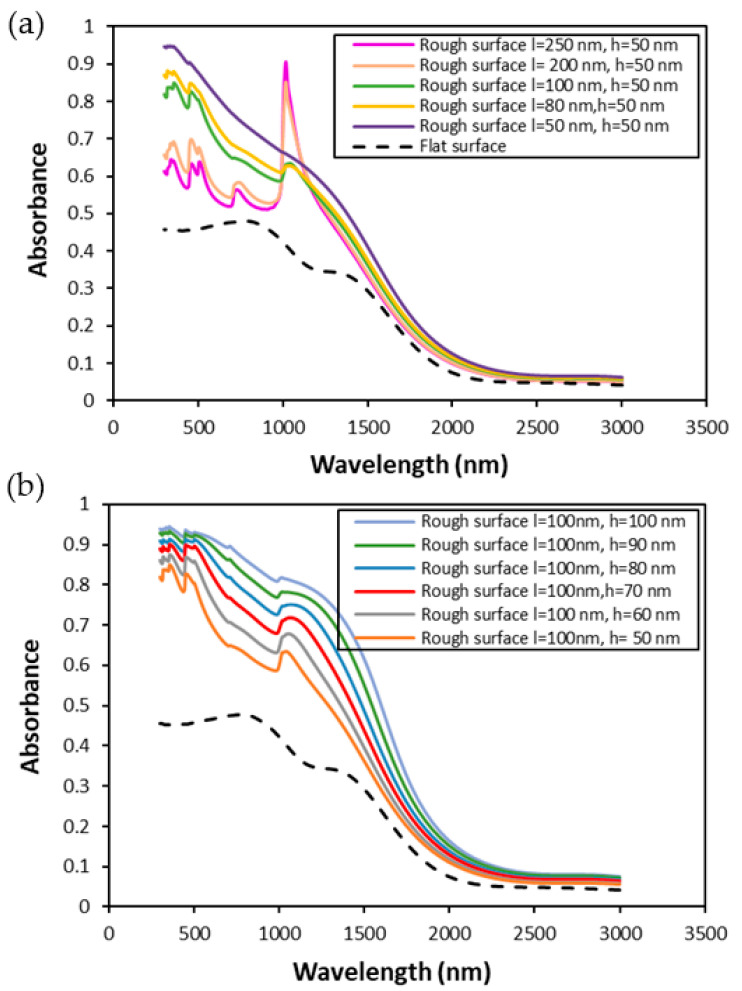
The effect of (**a**) correlation length and (**b**) RMS height in the absorption spectra of an isotropic random rough surface of Tungsten.

In Figure 5a, the absorption spectra of various surfaces with different correlation lengths at a fixed RMS height of h = 50 nm are given. As shown in Figure 5a, increasing the roughness by reducing the correlation length, which increases the slope of the surface facets at a fixed RMS height, enhances the absorption of Tungsten. A similar effect can be observed in Figure 5b, where the absorption spectra is given for various heights at a fixed correlation length of l = 100 nm. Both Figure 5a,b shows no significant difference between textures with varying l and h at longer wavelengths with decreasing the correlation length in the isotropic case. The absorption increases in the VIS region, as shown in Figure 5a. As we increase the correlation length to values larger than 200 nm, a peak in the absorption spectra is observed. It is noted that when the correlation length becomes larger than λ/5, a peak resonance is observed in the absorption spectra. This effect intensifies by increasing the RMS height of rough texture, and the peak point in the absorption spectra reaches unity. When optical coupling happens, absorption peaks occur. The physical mechanism that increases the interaction of electrons in the media with polarized light can be explained as more efficient light trapping through curved surfaces and an increased optical path during the scattering processes. The correlation length in the optimal structures provides the effective slopes for light trapping and increases the optical path. Based on the optimized values obtained in Figure 5a,b, a correlation length of 200 nm and a minimum height of 100 nm is selected to have an absorption peak in a specified wavelength region. Our results indicate that the spectral reflection from the samples is sensitive to the height, the correlation length, and the anisotropy of the tungsten surfaces. The features in the spectral reflection distributions thus can be an interesting future perspective to retrieve information about the height, the correlation length, and the anisotropy of the samples. The cluster analysis techniques are particularly promising to retrieve these geometric parameters, as it was applied recently to discriminate the dimensions of metal nano disks [64].

**Figure 6:**
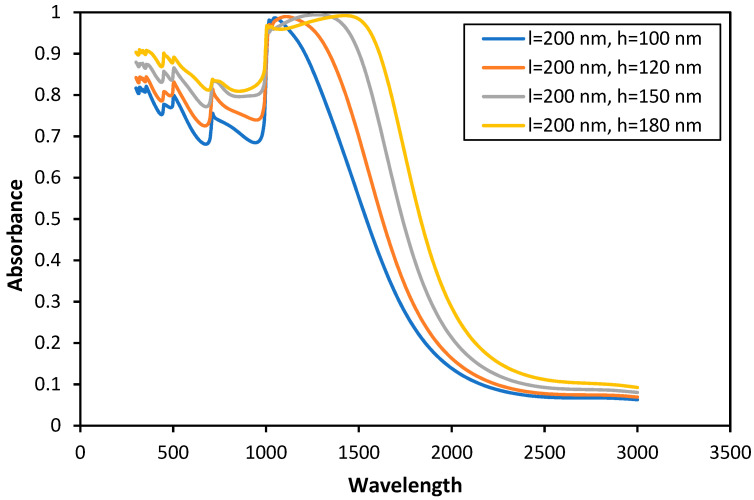
The RMS height effect on the bandwidth of absorbance.

The spectral distribution of the absorption of Tungsten surfaces can be influenced by the surface roughness by selectively changing the correlation length and RMS height of the random rough surface, as shown in Figure 6. In particular, the bandwidth of the spectral distribution can be greatly enhanced. This phenomenon can be used in the spectral selective absorbers, in which high absorption is needed at a specific wavelength. Based on the results in Figure 6, a surface with a correlation length of 200 nm and a RMS height of more than 100 nm show nearly 100% absorption at the wavelength in the range of 1000–1500 nm. A relatively narrowband absorption is observed for systems with a lower RMS height. Additionally, the VIS region has a lower spectral absorption. These characteristics are primarily due to the decreased optical coupling efficiency and narrow bandwidth of the resonances supported by the small surface peaks. Increasing the height of rough texture increases not only the absorptions in the VIS region but also the broad bandwidth of absorption. Furthermore, the near-unity absorption is accessible over a larger bandwidth. The underlying reason for this trend is that the rough surface height increases the degree of interaction between resonance and trapping light. Note that the higher the surface height, the more light trapping at long-wavelength. It should be noted that the absorption curve at low wavelengths (approximately up to 1000 nm) is flatter with a higher RMS height value. The results point out that the correlation length and RMS height can be used as controlling factors for a spectrally selective absorber. It is shown that for the wavelength in the range of 1000–1600 nm, a high absorption with a value around 0.95 is obtained just by the roughness of the surface without any additional coatings. In addition, an average of 47% improvement in the absorption is observed in the wavelength in the range of 300–1000 nm for the case of l = 200 nm and h = 180 nm in comparison to the flat surface. Simulation results show that increasing the RMS height with a selected correlation length increases the bandwidth of the absorption. This increase in absorption is caused by the coupling between the surface peaks’ resonant modes. For an RMS height of 180 nm, the absorption curve is over 85% in a wide wavelength range of 300–600 nm. At the same time, for the wavelengths of 1000–1600 nm, the absorption value is more than 95%. For higher RMS height values, the coupling between the surface peak’s resonance modes occurs in a wider range of wavelength, which cause a broader bandwidth of absorption.

**Figure 7:**
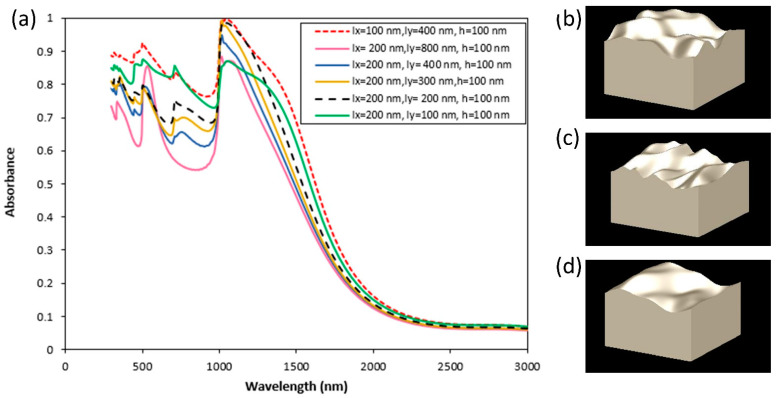
(**a**) The effect of correlation length for the W anisotropic rough surface. (**b**) Isotropic rough surface with l = 200 nm and h = 100 nm. (**c**) Anisotropic rough surface with l_x_ = 100 nm, l_y_ = 400 nm, and h = 100 nm. (**d**) Anisotropic rough surface with l_x_ = 200 nm, l_y_ = 400 nm, and h = 100 nm.

In the next step, inspired by grating couplers to control the light-matter interaction at material surfaces, we investigated the effect of anisotropic rough surfaces. Anisotropic surface roughness is obtained by a Gaussian surface with two different correlation lengths along x, y, marked as $l_{x}$, $l_{y}$, respectively. Figure 7 illustrates the absorption spectra for various anisotropic surfaces. The results in Figure 7 indicate that the absorption of the anisotropic rough surface is smaller than the isotropic rough surface with the same correlation length (l_x_ = 200 nm, Figure 7b) in the x-direction, which is shown with a black dash line in Figure 7a. It indicates that with the same correlation length in the x-direction, increasing the correlation length in the y-direction causes a drop in absorption values. However, smaller correlation length in the x-direction ($l_{x}$ = 100 nm) in the anisotropic case leads to more absorption in the VIS spectrum (red dash line in Figure 7a,c). Moreover, higher absorption can be obtained in the infrared region for the anisotropic case of l_x_ = 100 nm, l_y_ = 400 nm, and h = 100 nm. The absorption results of Tungsten anisotropic rough surface with l_x_ = 200 nm and l_y_ = 800 nm, which is illustrated in Figure 7d, show two clear resonance peaks in the spectrum, the first one is in 533 nm with 85% absorption, and the second one is 1082 nm with 87% absorption. In addition, the results show that anisotropic rough surfaces with lower correlation length in the x-direction can be used to obtain higher absorption in comparison to the isotropic rough surfaces with higher RMS height. In other words, at the same RMS height, anisotropic rough surfaces with lower correlation length can be used rather than the isotropic rough surfaces to have more absorption in VIS and near-infrared region.

An interesting observation in Figure 7 involves the two cases with a red dashed line (I_x_ = 100 nm, I_y_ = 400 nm) and a green solid line (I_x_ = 200 nm, I_y_ = 100 nm) with h = 100 nm. At an initial glance, even though the green solid line seems rougher than the red one, the red dashed line has higher absorption. The main reason for this is the polarization direction of the incident electric field and how it interacts with the anisotropic surfaces. In this case, the incident electric field is polarized in the x-direction. As a result, the x-polarized incident field is more sensitive to the roughness in the x-direction. In this case, the red dashed line is rougher in the x-direction (l_x_ = 100 nm) compared to the green solid line (l_x_ = 200 nm). This leads to a stronger absorption of the x-polarized incident field, even though the overall roughness of the green solid line is larger. An important consequence for anisotropic rough surfaces is that the roughness in the direction of polarization is more important than the overall roughness.

**Figure 8:**
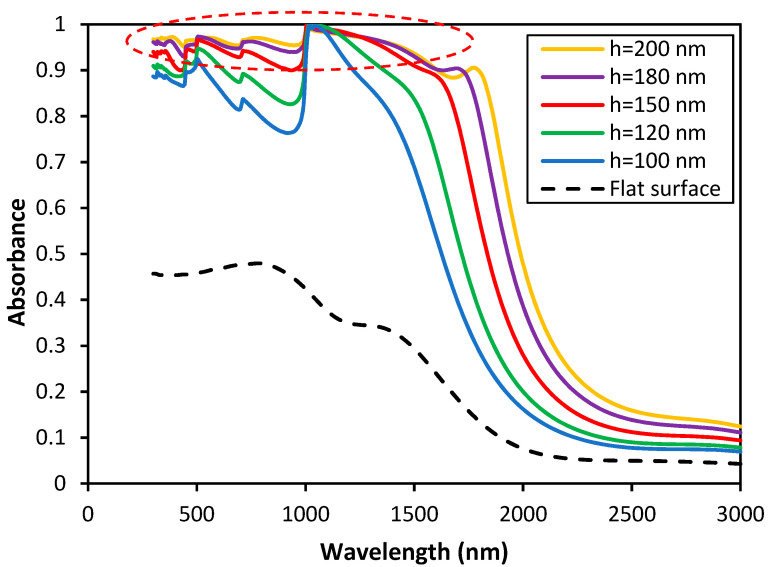
The RMS height effect on the absorbance of the anisotropic rough surface of Tungsten with l_x_ = 100 nm and l_y_ = 400 nm.

In Figure 8, the effect of RMS height on the spectral absorption is investigated. As mentioned before, for isotropic rough surfaces with increasing the RMS height, the absorption bandwidth increases. It is observed that starting from λ = 1000 nm, the perfect absorption spectrum is continuously broadened. For anisotropic random rough surfaces, a broad bandwidth is obtained with increasing the RMS height of the surface. This broad spectral region starts from λ = 300 nm and continues up to λ = 1500 nm. It is shown that for the case of the anisotropic rough surface with l_x_ = 100 nm and l_y_ = 400 nm and RMS height of 150 nm, the absorption is more than 90%. While taking this into account, the absorption curve has a maximum in the wavelength range of 300 to 1700 nm and covers the visible and near-infrared region. Moreover, for the RMS height of 180 nm the absorption reaches more than 95%, which is a significant improvement in comparison to a smooth surface. Broad absorption spectra were previously reported in the literature, but in all cases another material coating was added on to Tungsten to achieve such high absorptions [1,53]. We report such broad absorption spectra by surface modifications. Moreover, in comparison to the flat Tungsten surface, the absorption enhanced around 52% in the VIS region and approximately 80% for larger wavelengths from 1500–2000 nm.

**Figure 9:**
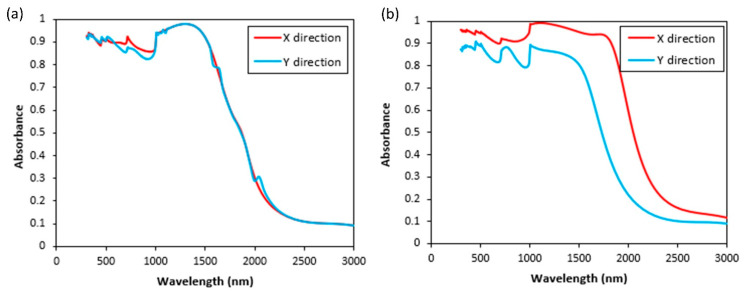
Polarization direction effect on the absorption spectrum for (**a**) isotropic surface (l = 200 nm, h = 180 nm). (**b**) Anisotropic surface (l_x_ = 100 nm, l_y_ = 400 nm, and h = 180 nm).

As mentioned earlier, a plane wave is used to illuminate the structures in our simulations. Moreover, the electric field is parallel to the surface and polarized in the x-direction and k vector is perpendicular to the structure. The polarization direction and angle of incidence are two other factors in studying scattering from a random rough surface. In this regard, the polarization direction in x- and y-direction corresponding to different wavelengths is plotted in Figure 9 for isotropic and anisotropic surfaces. For isotropic structures, changing the polarization does not affect the absorption spectrum as can be seen from Figure 9a, since the incident wave interacts similarly with features in both directions for the isotropic case. However, changing the polarization affects the results for the anisotropic case. As shown in Figure 9b, polarization in the y-direction will cause a reduction in absorption values for the anisotropic surface in this case.

**Figure 10:**
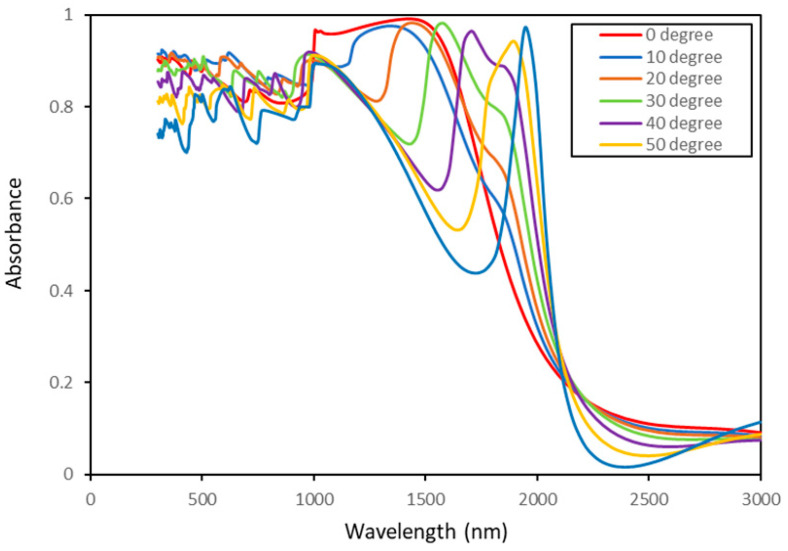
Absorbance spectrum regarding different incidence angles for an isotropic random rough surface.

Figure 10 illustrates the absorption spectrum for various incidence angles ranging from 0 to 60 degrees for an isotropic rough surface with l = 200 nm and h = 180 nm. As studied in the literature [65], the incident angle is an important factor in studying the light scattering from a metal rough surface. To show the importance of incident angle some studies have been done to introduce a structure to have a broadband absorption in most incident angles [66,67]. For the structure introduced in this study as a selective absorber, it can be seen from the figure that only in 0 degrees of incident we have a broadband absorption (red line). Moreover, by increasing the degrees of incident the peak band becomes narrow, but redshifts to longer wavelength. In addition, the overall absorption decreases when the incidence angle increases. Especially in the infrared range, a significant decrease in absorption is observed by increasing the incidence angle for this particular surface geometry.

## 4. Conclusions

In summary, we demonstrated a spectrally selective absorber surface made of Tungsten using an anisotropic Gaussian rough surface for high-temperature applications. It is shown that the rough surface enhances the absorption spectra both in the visible and near-infrared spectral regions. For isotropic random rough surfaces, it is shown that at correlation lengths higher than 200 nm, a peak started to appear in the absorption curve around the wavelength of 1000 nm. Increasing the RMS height leads to broadening the bandwidth of near-unity absorption for the 1000–1600 nm region. Our investigation shows that using the anisotropic random rough surface design yields even more interesting results in terms of the absorption spectrum. Larger than 90% absorption is obtained for the optimized anisotropic random rough surface for the spectral range from 300–1700 nm, suggesting a perfect absorption bandwidth up to 1700 nm in the ultraviolet visible and near-infrared region. These optimized random nanostructures provide multiple resonant modes, which introduce strong optical coupling and result in a broadening of the high absorption spectrum region in the range of 300–1700 nm. The proposed spectrally selective absorber in this study eliminates the need for adding extra layers or nanoparticles to act as spectral selective emitters. Moreover, this technique avoids inserting additional surface thickness by adding coatings of different materials. These findings can be used in tailoring the broadband spectrum for different applications in which the surfaces are exposed to high temperatures, such as solar and aerospace applications.

## Data Availability

The data that support the findings of this study are available from the corresponding author upon reasonable request.

## References

[1] Han X., He K., He Z., Zhang Z. (2017). Tungsten-based highly selective solar absorber using simple nanodisk array. Opt. Express.

[2] Sibin K.P., John S., Barshilia H.C. (2015). Control of thermal emittance of stainless steel using sputtered tungsten thin films for solar thermal power applications. Sol. Energy Mater. Sol. Cells.

[3] Cao F., Kraemer D., Sun T., Lan Y., Chen G., Ren Z. (2015). Enhanced Thermal Stability of W-Ni-Al2O3 Cermet-Based Spectrally Selective Solar Absorbers with Tungsten Infrared Reflectors. Adv. Energy Mater..

[4] Silva-Oelker G., Jerez-Hanckes C., Fay P. (2019). High-temperature tungsten-hafnia optimized selective thermal emitters for thermophotovoltaic applications. J. Quant. Spectrosc. Radiat. Transf..

[5] Khan A., Elliman R., Corr C., Lim J.J.H., Forrest A., Mummery P., Evans L.M. (2016). Effect of rhenium irradiations on the mechanical properties of tungsten for nuclear fusion applications. J. Nucl. Mater..

[6] Rieth M., Dudarev S.L., De Vicente S.M.G., Aktaa J., Ahlgren T., Antusch S., Armstrong D.E.J., Balden M., Baluc N., Barthe M.-F. (2013). Recent progress in research on tungsten materials for nuclear fusion applications in Europe. J. Nucl. Mater..

[7] Marinelli G., Martina F., Lewtas H., Hancock D., Mehraban S., Lavery N., Ganguly S., Williams S. (2019). Microstructure and thermal properties of unalloyed tungsten deposited by Wire+ Arc Additive Manufacture. J. Nucl. Mater..

[8] Liu R., Wang Z., Sparks T., Liou F., Newkirk J. (2017). Aerospace applications of laser additive manufacturing. Laser Additive Manufacturing.

[9] Ungaro C., Gray S.K., Gupta M.C. (2013). Black tungsten for solar power generation. Appl. Phys. Lett..

[10] Wang H., Wang L. (2013). Perfect selective metamaterial solar absorbers. Opt. Express.

[11] Song J., Si M., Cheng Q., Luo Z. (2016). Two-dimensional trilayer grating with a metal/insulator/metal structure as a thermophotovoltaic emitter. Appl. Opt..

[12] Zhang H., Luo M., Zhou Y., Ji Y., Chen L. (2020). Ultra-broadband, polarization-independent, wide-angle near-perfect absorber incorporating a one-dimensional meta-surface with refractory materials from UV to the near-infrared region. Opt. Mater. Express.

[13] Rakić A.D., Djurišić A.B., Elazar J.M., Majewski M.L. (1998). Optical properties of metallic films for vertical-cavity optoelectronic devices. Appl. Opt..

[14] Rephaeli E., Fan S. (2008). Tungsten black absorber for solar light with wide angular operation range. Appl. Phys. Lett..

[15] Rinnerbauer V., Lenert A., Bierman D.M., Yeng Y.X., Chan W.R., Geil R.D., Senkevich J.J., Joannopoulos J.D., Wang E.N., Soljačić M. (2014). Metallic photonic crystal absorber-emitter for efficient spectral control in high-temperature solar thermophotovoltaics. Adv. Energy Mater..

[16] Celanovic I., Jovanovic N., Kassakian J. (2008). Two-dimensional tungsten photonic crystals as selective thermal emitters. Appl. Phys. Lett..

[17] Wang H., Sivan V.P., Mitchell A., Rosengarten G., Phelan P., Wang L. (2015). Highly efficient selective metamaterial absorber for high-temperature solar thermal energy harvesting. Sol. Energy Mater. Sol. Cells.

[18] Khodasevych I.E., Wang L., Mitchell A., Rosengarten G. (2015). Micro and nanostructured surfaces for selective solar absorption. Adv. Opt. Mater..

[19] Gupta M.C., Ungaro C., Foley IV J.J., Gray S.K. (2018). Optical nanostructures design, fabrication, and applications for solar/thermal energy conversion. Sol. Energy.

[20] Shah A.A., Gupta M.C. (2013). Spectral selective surfaces for concentrated solar power receivers by laser sintering of tungsten micro and nano particles. Sol. Energy Mater. Sol. Cells.

[21] Kecebas M.A., Menguc M.P., Kosar A., Sendur K. (2020). Spectrally selective filter design for passive radiative cooling. JOSA B.

[22] Gupta M.C., Carlson D.E. (2015). Laser processing of materials for renewable energy applications. MRS Energy Sustain..

[23] Jeong H.-J., Kim Y.-C., Lee S.K., Yun J.-H., Jang J.-H. (2019). Enhanced spectral response of CIGS solar cells with anti-reflective subwavelength structures and quantum dots. Sol. Energy Mater. Sol. Cells.

[24] Benamira A., Pattanaik S. (2020). Application of the Transfer Matrix Method to Anti-reflective Coating Rendering. Proceedings of the Computer Graphics International Conference.

[25] Hobbs D.S. (2012). Random Texture Anti-Reflection Optical Surface Treatment. U.S. Patent.

[26] Cho C., Kim H., Jeong S., Baek S.-W., Seo J.-W., Han D., Kim K., Park Y., Yoo S., Lee J.-Y. (2013). Random and V-groove texturing for efficient light trapping in organic photovoltaic cells. Sol. Energy Mater. Sol. Cells.

[27] Goulas A., Zhang S., McGhee J.R., Cadman D.A., Whittow W.G., Vardaxoglou J.C., Engstrøm D.S. (2020). Fused filament fabrication of functionally graded polymer composites with variable relative permittivity for microwave devices. Mater. Des..

[28] Carbonaro C.M., Corpino R., Salis M., Mocci F., Thakkar S.V., Olla C., Ricci P.C. (2019). On the emission properties of carbon dots: Reviewing data and discussing models. C.

[29] Chen C., Wang J., Gao Y. (2021). Wavelength-Dependent Nonlinear Absorption in Palladium Nanoparticles. Appl. Sci..

[30] Baffou G., Cichos F., Quidant R. (2020). Applications and challenges of thermoplasmonics. Nat. Mater..

[31] Zograf G.P., Petrov M.I., Makarov S.V., Kivshar Y.S. (2021). All-dielectric thermonanophotonics. arXiv.

[32] Zograf G.P., Petrov M.I., Zuev D.A., Dmitriev P.A., Milichko V.A., Makarov S.V., Belov P.A. (2017). Resonant nonplasmonic nanoparticles for efficient temperature-feedback optical heating. Nano Lett..

[33] Gandolfi M., Crut A., Medeghini F., Stoll T., Maioli P., Vallée F., Banfi F., Del Fatti N. (2018). Ultrafast thermo-optical dynamics of plasmonic nanoparticles. J. Phys. Chem. C.

[34] Ishii S., Sugavaneshwar R.P., Nagao T. (2016). Titanium nitride nanoparticles as plasmonic solar heat transducers. J. Phys. Chem. C.

[35] Li M., Lohmuller T., Feldmann J. (2015). Optical injection of gold nanoparticles into living cells. Nano Lett..

[36] Kristensen A., Yang J.K.W., Bozhevolnyi S.I., Link S., Nordlander P., Halas N.J., Mortensen N.A. (2016). Plasmonic colour generation. Nat. Rev. Mater..

[37] Gandolfi M., Banfi F., Glorieux C. (2020). Optical wavelength dependence of photoacoustic signal of gold nanofluid. Photoacoustics.

[38] Celebrano M., Rocco D., Gandolfi M., Zilli A., Rusconi F., Tognazzi A., Mazzanti A., Ghirardini L., Pogna E.A.A., Carletti L. (2021). Optical tuning of dielectric nanoantennas for thermo-optically reconfigurable nonlinear metasurfaces. Opt. Lett..

[39] Tian Y., Ghanekar A., Ricci M., Hyde M., Gregory O., Zheng Y. (2018). A review of tunable wavelength selectivity of metamaterials in near-field and far-field radiative thermal transport. Materials.

[40] Wu Z., Xue W., Liu Y., Wei D., Wang J., Yin L., Wang Y., Liu X., Zhang Q., Cao F. (2020). Toward versatile applications via tuning transition wavelength of the WTa-SiO2 based spectrally selective absorber. Sol. Energy.

[41] Calisgan S.D., Villanueva-Lopez V., Rajaram V., Qian Z., Kang S., Hernandez-Rivera S.P., Rinaldi M. Spectroscopic chemical sensing based on narrowband MEMS resonant infrared detectors. Proceedings of the 2018 IEEE SENSORS.

[42] Zhao B., Hu M., Ao X., Xuan Q., Pei G. (2020). Spectrally selective approaches for passive cooling of solar cells: A review. Appl. Energy.

[43] Khosroshahi F.K., Ertürk H., Mengüç M.P. (2017). Optimization of spectrally selective Si/SiO2 based filters for thermophotovoltaic devices. J. Quant. Spectrosc. Radiat. Transf..

[44] Dan A., Jyothi J., Chattopadhyay K., Barshilia H.C., Basu B. (2016). Spectrally selective absorber coating of WAlN/WAlON/Al2O3 for solar thermal applications. Sol. Energy Mater. Sol. Cells.

[45] Mehrabi S., Rezaei M.H., Zarifkar A. (2019). Ultra-broadband solar absorber based on multi-layer TiN/TiO 2 structure with near-unity absorption. JOSA B.

[46] Chattopadhyay S., Huang Y.F., Jen Y.J.A. (2010). Ganguly, KH Chen and LC Chen. Mater. Sci. Eng. R.

[47] Tan H., Santbergen R., Smets A.H.M., Zeman M. (2012). Plasmonic light trapping in thin-film silicon solar cells with improved self-assembled silver nanoparticles. Nano Lett..

[48] Bergström D., Powell J., Kaplan A.F.H. (2008). The absorption of light by rough metal surfaces—A three-dimensional ray-tracing analysis. J. Appl. Phys..

[49] De Castro C.P., Luković M., Andrade R.F.S., Herrmann H.J. (2017). The influence of statistical properties of Fourier coefficients on random Gaussian surfaces. Sci. Rep..

[50] Niu C., Zhu T., Lv Y. (2019). Influence of Surface Morphology on Absorptivity of Light-Absorbing Materials. Int. J. Photoenergy.

[51] Sai H., Kanamori Y. (2003). Spectrally selective thermal radiators and absorbers with periodic microstructured surface for high-temperature applications. Microscale Thermophys. Eng..

[52] Cao L., Sendur K. (2019). Surface Roughness Effects on the Broadband Reflection for Refractory Metals and Polar Dielectrics. Materials.

[53] Raza A., Alketbi A.S., Devarapalli R., Li H., Zhang T. (2020). Refractory Ultrathin Nanocomposite Solar Absorber with Superior Spectral Selectivity and Thermal Stability. Adv. Opt. Mater..

[54] Lumerical F. Solutions 2016. http://www.lumerical.com/.

[55] Palik E.D. (1998). Handbook of Optical Constants of Solids.

[56] Liu B., Xia X., Sun C. (2018). Scattering properties of solid rough surface of nickel skeleton. Infrared Phys. Technol..

[57] Liu Z., Liu G., Huang Z., Liu X., Fu G. (2018). Ultra-broadband perfect solar absorber by an ultra-thin refractory titanium nitride meta-surface. Sol. Energy Mater. Sol. Cells.

[58] Shimizu M., Yugami H. (2011). Thermal radiation control by surface gratings as an advanced cooling system for electronic devices. J. Therm. Sci. Technol..

[59] Sai H., Yugami H., Kanamori Y., Hane K. (2003). Solar selective absorbers based on two-dimensional W surface gratings with submicron periods for high-temperature photothermal conversion. Sol. Energy Mater. Sol. Cells.

[60] Amiri I.S., Sorger V.J., Yupapin P. (2019). Zinc Oxide nanowire gratings for light absorption control through polarization manipulation. Phys. E Low-Dimens. Syst. Nanostruct..

[61] Wojciechowski S., Twardowski P., Chwalczuk T. (2014). Surface roughness analysis after machining of direct laser deposited tungsten carbide. Proc. J. Phys. Conf Ser..

[62] Maleki E., Bagherifard S., Bandini M., Guagliano M. (2020). Surface post-treatments for metal additive manufacturing: Progress, challenges, and opportunities. Addit. Manuf..

[63] Hatefi S., Abou-El-Hossein K. (2020). Review of single-point diamond turning process in terms of ultra-precision optical surface roughness. Int. J. Adv. Manuf. Technol..

[64] Ronchi A., Sterzi A., Gandolfi M., Belarouci A., Giannetti C., Del Fatti N., Banfi F., Ferrini G. (2021). Discrimination of nano-objects via cluster analysis techniques applied to time-resolved thermo-acoustic microscopy. Ultrasonics.

[65] Voti R.L., Leahu G.L., Gaetani S., Sibilia C., Violante V., Castagna E., Bertolotti M. (2009). Light scattering from a rough metal surface: Theory and experiment. JOSA B.

[66] Larciprete M.C., Centini M., Voti R.L., Bertolotti M., Sibilia C. (2014). Polarization insensitive infrared absorbing behaviour of one-dimensional multilayer stack: A fractal approach. Opt. Express.

[67] Larciprete M.C., Centini M., Voti R.L., Bertolotti M., Sibilia C. (2016). Metallic oriented nanowires films for infrared radiation manipulation. Appl. Phys. A.

